# Substitution of l-Tryptophan by **α**-Methyl-l-Tryptophan in ^177^Lu-RM2 Results in ^177^Lu-AMTG, a High-Affinity Gastrin-Releasing Peptide Receptor Ligand with Improved In Vivo Stability

**DOI:** 10.2967/jnumed.121.263323

**Published:** 2022-09

**Authors:** Thomas Günther, Sandra Deiser, Veronika Felber, Roswitha Beck, Hans-Jürgen Wester

**Affiliations:** Chair of Pharmaceutical Radiochemistry, Technical University of Munich, Garching, Germany

**Keywords:** AMTG, GRPR, RM2, prostate cancer, increased metabolic stability, NeoBOMB1

## Abstract

Theranostic applications targeting the gastrin-releasing peptide receptor (GRPR) have shown promising results. When compared with other peptide ligands for radioligand therapy, the most often used GRPR ligand, DOTA-Pip^5^-d-Phe^6^-Gln^7^-Trp^8^-Ala^9^-Val^10^-Gly^11^-His^12^-Sta^13^-Leu^14^-NH_2_ (RM2), may be clinically impacted by limited metabolic stability. With the aim of improving the metabolic stability of RM2, we investigated whether the metabolically unstable Gln^7^-Trp^8^ bond within the pharmacophore of RM2 can be stabilized via substitution of l-Trp^8^ by α-methyl-l-tryptophan (α-Me-l-Trp) and whether the corresponding DOTAGA analog might also be advantageous. A comparative preclinical evaluation of ^177^Lu-α-Me-l-Trp^8^-RM2 (^177^Lu-AMTG) and its DOTAGA counterpart (^177^Lu-AMTG2) was performed using ^177^Lu-RM2 and ^177^Lu-NeoBOMB1 as reference compounds. **Methods:** Peptides were synthesized by solid-phase peptide synthesis and labeled with ^177^Lu. Lipophilicity was determined at pH 7.4 (log*D*_7.4_). Receptor-mediated internalization was investigated on PC-3 cells (37°C, 60 min), whereas GRPR affinity (half-maximal inhibitory concentration) was determined on both PC-3 and T-47D cells. Stability toward peptidases was examined in vitro (human plasma, 37°C, 72 ± 2 h) and in vivo (murine plasma, 30 min after injection). Biodistribution studies were performed at 24 h after injection, and small-animal SPECT/CT was performed on PC-3 tumor–bearing mice at 1, 4, 8, 24, and 28 h after injection. **Results:** Solid-phase peptide synthesis yielded 9%–15% purified labeling precursors. ^177^Lu labeling proceeded quantitatively. Compared with ^177^Lu-RM2, ^177^Lu-AMTG showed slightly improved GRPR affinity, a similar low internalization rate, slightly increased lipophilicity, and considerably improved stability in vitro and in vivo. In vivo, ^177^Lu-AMTG exhibited the highest tumor retention (11.45 ± 0.43 percentage injected dose/g) and tumor-to-blood ratio (2,702 ± 321) at 24 h after injection, as well as a favorable biodistribution profile. As demonstrated by small-animal SPECT/CT imaging, ^177^Lu-AMTG also revealed a less rapid clearance from tumor tissue. Compared with ^177^Lu-AMTG, ^177^Lu-AMTG2 did not show any further benefits. **Conclusion:** The results of this study, particularly the superior metabolic stability of ^177^Lu-AMTG, strongly recommend a clinical evaluation of this novel GRPR-targeted ligand to investigate its potential for radioligand therapy of GRPR-expressing malignancies.

## Visual Abstract

**Figure:**
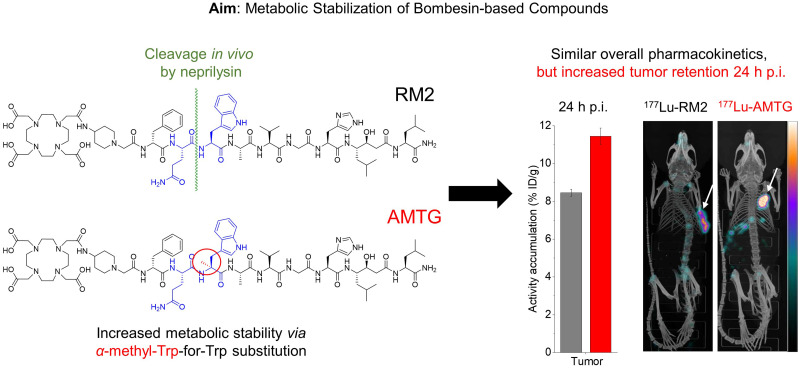

Radioligand therapy has emerged as a powerful alternative to conventional treatment options in oncology. This emergence can be attributed mainly to the success of DOTATOC- and DOTATATE-based theranostics in the case of neuroendocrine tumors and to prostate-specific membrane antigen (PSMA)–targeted inhibitors in the case of prostate cancer (*1**,**2*). In view of the overexpression of the gastrin-releasing peptide receptor (GRPR, bombesin-2 receptor) at a high density and frequency already in early stages of prostate cancer (∼5,000 disintegrations/min [dpm]/mg of tissue, with >2,000 dpm/mg being considered clinically relevant) and breast cancer (∼10,000 dpm/mg), GRPR has been identified as a promising target for both cancer types (*3**,**4*).

In a recent study, 50 patients with biochemically recurrent prostate cancer were examined with either ^68^Ga-PSMA11 or ^18^F-DCFPyL PET/CT and additionally with ^68^Ga-RM2 PET/MRI (where RM2 is DOTA-Pip^5^-d-Phe^6^-Gln^7^-Trp^8^-Ala^9^-Val^10^-Gly^11^-His^12^-Sta^13^-Leu^14^-NH_2_). Thirty-six lesions were visible only with ^68^Ga-PSMA11/^18^F-DCFPyL PET/CT, and 7 only with ^68^Ga-RM2 PET/MRI, which again suggests a complementary role for GRPR- and PSMA-targeted theranostics (*5*). Moreover, estrogen receptor–rich breast cancer (estrogen receptor being expressed in over 80% of all breast cancers), in particular, shows high GRPR expression, which is retained in 95% of nodal metastases (*6**,**7*). Not surprisingly, successful high-contrast PET imaging of breast cancer using ^68^Ga-NOTA-RM26 or ^68^Ga-RM2 has already been described (*8**,**9*).

The 2 most promising GRPR-targeted radiopharmaceuticals, ^68^Ga-RM2 and ^68^Ga-NeoBOMB1, have already shown favorable initial results and are being assessed in phase 1 and 2 clinical studies (*10**–**13*). A first-in-humans study on ^177^Lu-RM2 in PSMA-negative/GRPR-positive prostate cancer revealed encouraging dosimetry data (*14*). Nevertheless, limited metabolic stability of some bombesin derivatives, such as RM2, is well known and caused mainly by the neutral endopeptidase (Enzyme Commission no. 3.4.24.11), which reportedly cleaves linear peptides at the *N* terminus side of hydrophobic amino acids (*15*). Incubation of ^177^Lu-labeled DOTA-4-aminobenzoyl-Gln^7^- Trp^8^-Ala^9^- Val^10^- Gly^11^- His^12^- Leu^13^-Met^14^-NH_2_ (AMBA) in murine and human plasma in vitro revealed several cleavage sites, especially at the *C* terminus and the Gln^7^-Trp^8^ site (*16*). Similar observations were made in 5 healthy patients, as the administered ^68^Ga-RM2 showed only 19% intact tracer in blood at 65 min after injection (*17*). Considering this rather small fraction of intact compound early after injection, a metabolically stabilized RM2 analog could result in improved tumor uptake, tumor retention, and thus tumor dose. In recent years, many groups developed bombesin analogs that were modified at the *C* or *N* termini, but not within the pharmacophoric sequence (Gln^7^-Trp^8^-Ala^9^-Val^10^-Gly^11^-His^12^) (*18**–**22*).

As we hypothesized that the use of statine (i.e., Sta^13^) at the *C* terminus of RM2 and its derivatives would result in sufficient metabolic stabilization at this part of the molecule, we concluded that further improvements might be possible by stabilizing the Gln^7^-Trp^8^ sequence. For this purpose, we substituted α-methyl-l-tryptophan (α-Me-l-Trp) for l-Trp^8^ in ^177^Lu-RM2 and its DOTAGA analog (Fig. 1) and evaluated these novel compounds alongside the potent reference ligands ^177^Lu-RM2 and ^177^Lu-NeoBOMB1. The comparative preclinical evaluation comprises affinity studies (half-maximal inhibitory concentration, or *IC*_50_) on PC-3 and T-47D cells, quantification of receptor-mediated internalization on PC-3 cells, determination of lipophilicity at pH 7.4 (log*D*_7.4_), investigations of stability against peptidases in vitro in human plasma and in vivo in plasma and urine of mice, and biodistribution studies on PC-3 tumor–bearing mice.

**FIGURE 1.**
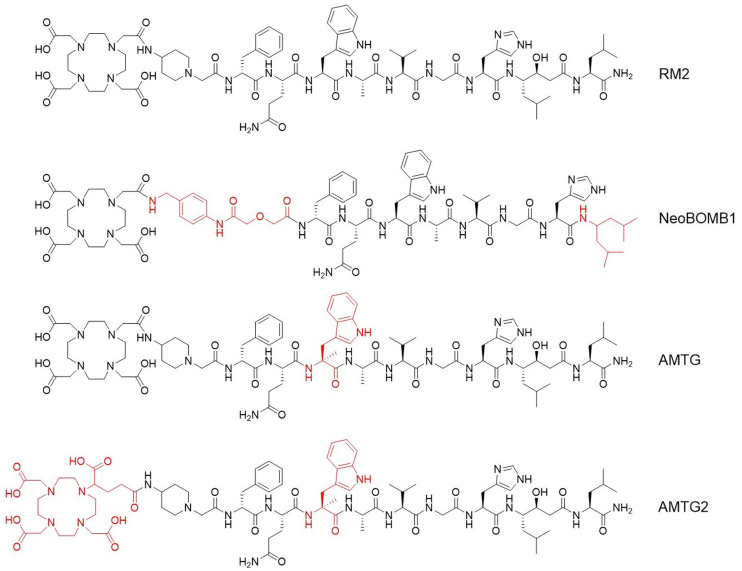
Chemical structure of RM2 and its α-Me-l-Trp (α-Me-l-Trp^8^) modified derivatives AMTG and AMTG2, as well as reference ligand NeoBOMB1. Structural differences from RM2 are highlighted in red.

## MATERIALS AND METHODS

### Chemical Synthesis and Labeling Procedures

RM2 derivatives were prepared via standard Fmoc-based solid-phase peptide synthesis using *H*-Rink amide ChemMatrix resin (35- to 100-mesh particle size, 0.4–0.6 mmol/g loading; Merck KGaA). NeoBOMB1 was synthesized according to a reported procedure (*20*) and purified by reversed-phase high-performance liquid chromatography (RP-HPLC).

Both ^nat^Lu and ^177^Lu labeling was according to a modified procedure (*23*). The radiolabeled reference, 3-^125^I-d-Tyr^6^-MJ9 (Supplemental Figs. 1 and 2; supplemental materials are available at http://jnm.snmjournals.org), was prepared according to a reported procedure (*24*). A detailed description of the synthesis, labeling, and characterization of RM2 and its analogs is provided in Supplemental Figures 3–12.

### In Vitro Experiments

A detailed description of all cell-based experiments is provided in the supplemental materials.

#### Affinity Determinations (IC_50_) and Internalization Studies

Competitive binding studies were performed on both PC-3 and T-47D cells (1.5 × 10^5^ cells in 1 mL/well) via incubation at room temperature for 2 h using 3-^125^I-d-Tyr^6^-MJ9 (0.2 nM/well) as a radiolabeled reference (*n* = 3). Internalization studies of the ^177^Lu-labeled conjugates (1.0 nM/well) were performed on PC-3 cells (1.5 × 10^5^ cells in 1 mL/well) at 37°C for 1 h (*n* = 6). Data were corrected for nonspecific binding (competition by 10^−3^ M ^nat^Lu-RM2).

#### Determination of Lipophilicity (*n*-Octanol–Phosphate-Buffered Saline Distribution Coefficient, logD_7.4_)

Approximately 1 MBq of the ^177^Lu-labeled compound was dissolved in 1 mL of phosphate-buffered saline (pH 7.4) and *n*-octanol (*v*/*v* = 1/1). After stirring for 3 min at room temperature in a vortex mixer and subsequent centrifugation at 9,000 rpm for 5 min (Biofuge 15; Heraeus Sepatech GmbH), 200-μL aliquots of both layers were measured separately in a *γ*-counter. The experiment was repeated at least 5 times.

#### In Vitro Stability Studies

Metabolic stability in vitro was determined through a procedure published by Linder et al. that was slightly modified (*16*). Immediately after labeling, 200 μL of human plasma were added and the mixture was incubated at 37°C for 72 ± 2 h. Proteins were precipitated by treatment with ice-cold EtOH (150 μL) and ice-cold MeCN (450 μL), followed by centrifugation (13,000 rpm, 20 min). The supernatants were decanted and further centrifuged (13,000 rpm, 10 min) using a Costar Spin-X (Corning) centrifuge tube filter (0.45 μm). The filtrated plasma samples were analyzed using radio–RP-HPLC.

### In Vivo Experiments

All animal experiments were conducted in accordance with general animal welfare regulations in Germany (German animal protection act, as amended on 18.05.2018, article 141 G v. 29.3.2017 I 626, approval ROB-55.2-2532.Vet_02-18-109) and the institutional guidelines for the care and use of animals.

#### In Vivo Stability Studies

Approximately 30–40 MBq (1 nmol, 150 μL) of the ^177^Lu-labeled compounds were injected into the tail vein of anesthetized CB17-SCID mice (*n* = 3). After euthanasia at 30 min after injection, blood and urine samples were collected. Blood proteins were precipitated by treatment with ice-cold MeCN (*v*/*v* = 1/1), followed by centrifugation (13,000 rpm, 20 min). The supernatants were decanted and further centrifuged (13,000 rpm, 10 min) using a Costar Spin-X centrifuge tube filter (0.45 μm). The filtrated plasma samples and the urine samples were analyzed using radio–RP-HPLC.

#### Biodistribution and Small-Animal SPECT/CT Imaging Studies

A detailed description of tumor inoculation is provided in the supplemental materials. Approximately 2–5 MBq (100 pmol, 150 μL) of the ^177^Lu-labeled GRPR ligands were injected into the tail vein of anesthetized (2% isoflurane) PC-3 tumor–bearing mice (biodistribution, *n* = 4; imaging, *n* = 1).

For biodistribution studies, organs were removed, weighed, and measured in a *γ*-counter (Perkin Elmer) after euthanasia at 24 h after injection.

Imaging studies were performed on a MILabs VECTor^4^ small-animal SPECT/PET/optical imaging/CT device (MILabs). Data were reconstructed using the MILabs Rec software (version 10.02) and a pixel-based algorithm (similarity-regulated ordered-subsets expectation maximization), followed by data analysis using PMOD software (version 4.0; PMOD Technologies LLC). Static images were recorded at t = 1, 4, 8, 24, and 28 h after injection with an acquisition time of t + (45–60 min) using a high-energy general-purpose rat and mouse collimator and a stepwise multiplanar bed movement.

For all competition studies, 3.62 mg/kg (40 nmol) of ^nat^Lu-RM2 (10^−3^ M in phosphate-buffered saline) were coadministered.

## RESULTS

### Synthesis and Radiolabeling

Uncomplexed ligands were synthesized via standard Fmoc-based solid-phase peptide synthesis, yielding 9%–15% of each labeling precursor after purification by RP-HPLC (chemical purity > 98%, determined by RP-HPLC at λ = 220 nm). Complexation of all ligands with a 2.5-fold excess of ^nat^LuCl_3_ resulted in quantitative yields. The remaining free Lu^3+^ did not affect the cell-based assay in a brief competition study (Supplemental Fig. 13); thus, purification before affinity studies was dispensed with. ^125^I-iodination of d-Tyr^6^-MJ9 by means of the Iodo-Gen (Pierce Biotechnology, Inc.) method resulted in 3-^125^I-d-Tyr^6^-MJ9 with radiochemical yields of 33%–48% and radiochemical purities of more than 98% after RP-HPLC purification. ^177^Lu labeling of all compounds was performed manually, each resulting in quantitative radiochemical yields, radiochemical purities of more than 98%, and molar activities of 40 ± 10 GBq/μmol. All ^177^Lu-labeled ligands were used without further purification.

### In Vitro Characterization

**FIGURE 2.**
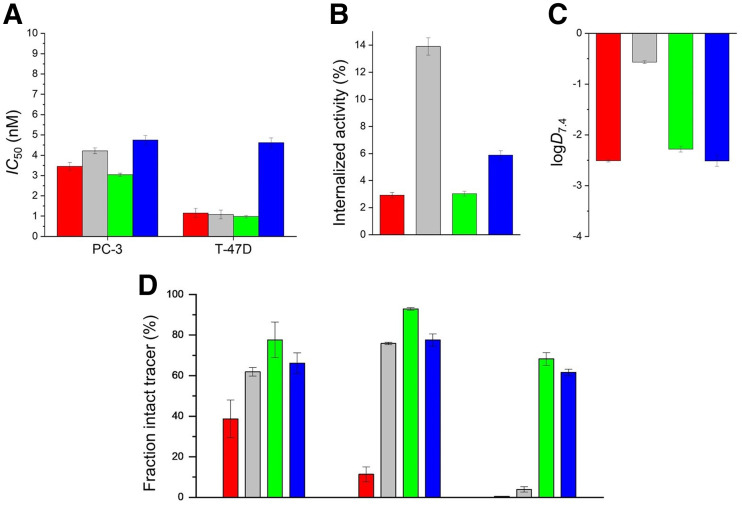
Preclinical data of ^nat/177^Lu-RM2 (red), ^nat/177^Lu-NeoBOMB1 (gray), ^nat/177^Lu-AMTG (green), and ^nat/177^Lu-AMTG2 (blue). (A) Affinity data on PC-3 and T-47D cells (1.5 × 10^5^ cells/mL/well) using 3-^125^I-d-Tyr^6^-MJ9 (0.2 nM/well) as radiolabeled reference (2 h, room temperature, Hanks’ balanced salt solution plus 1% bovine serum albumin [*v*/*v*]). (B) GRPR-mediated internalization (0.25 pmol/well) on PC-3 cells as percentage of applied activity (incubation at 37°C for 1 h, Dulbecco modified Eagle medium/F-12 plus 5% bovine serum albumin [*v*/*v*], 1.5 × 10^5^ cells/mL/well). Data were corrected for nonspecific binding (10^−3^ M ^nat^Lu-RM2). (C) Lipophilicity at pH 7.4 (log*D*_7.4_). (D) Metabolic stability in vitro in human plasma (left) (37°C, 72 ± 2 h; *n* = 4), and metabolic stability in vivo in murine plasma (middle) and murine urine (right) at 30 min after injection (*n* = 3). Data are expressed as mean ± SD. Metabolic stability of ^177^Lu-RM2 derivatives was determined in vitro and in vivo.

In vitro data on the examined bombesin-based ligands are summarized in Figure 2 and Supplemental Table 1. The cold counterpart of 3-^125^I-d-Tyr^6^-MJ9 showed an *IC*_50_ of 1.3 ± 0.4 nM on PC-3 cells. The ^nat^Lu-labeled compounds exhibited *IC*_50_ values in a range of 3.0–4.7 on PC-3 cells and 1.0–4.6 nM on T-47D cells. ^177^Lu-α-Me-l-Trp^8^-RM2 (^177^Lu-AMTG) and ^177^Lu-RM2 were internalized by PC-3 cells within 1 h in similar amounts (3.03% ± 0.18% vs. 2.92% ± 0.20%). ^177^Lu-DOTAGA-α-Me-l-Trp^8^-RM2 (^177^Lu-AMTG2) (5.88% ± 0.33%) and ^177^Lu-NeoBOMB1 (13.91% ± 0.64%) were taken up in higher amounts. Whereas the distribution coefficients (log*D*_7.4_) were quite similar for ^177^Lu-RM2 and its analogs (−2.3 to −2.5), ^177^Lu-NeoBOMB1 was considerably more lipophilic (−0.57 ± 0.03). The highest amounts of intact compound in vitro in human plasma were found for ^177^Lu-AMTG (77.7% ± 8.7%). Whereas ^177^Lu-AMTG2 and ^177^Lu-NeoBOMB1 exhibited only a slightly reduced stability in vitro (66.2% ± 5.1% vs. 61.9% ± 2.1%), only 38.7% ± 9.3% intact ^177^Lu-RM2 was found after incubation in human plasma at 37°C for 72 ± 2 h (Fig. 2; Supplemental Fig. 14).

### In Vivo Characterization

In vivo stability in murine plasma at 30 min after injection was highest for ^177^Lu-AMTG (92.9% ± 0.7% intact tracer). Again, slightly decreased metabolic stability was observed for ^177^Lu-NeoBOMB1 (75.9% ± 0.6%) and ^177^Lu-AMTG2 (77.6% ± 3.1%), whereas ^177^Lu-RM2 was quite unstable (11.4% ± 3.7%). In addition to these findings, ^177^Lu-AMTG and ^177^Lu-AMTG2 were excreted into the urine at 30 min after injection, predominantly as intact tracers (68.2% ± 3.1% and 61.6% ± 1.6%, respectively) (Fig. 2; Supplemental Figs. 15 and 16). Interestingly, after injection of ^177^Lu-RM2 and ^177^Lu-NeoBOMB1, radioactivity appeared in the urine almost quantitatively in the form of their metabolites (0.5% ± 0.1% vs. 3.9% ± 1.3% intact tracer).

Biodistribution studies on PC-3 tumor–bearing mice were performed at 24 h after injection (Table 1). ^177^Lu-RM2 and its derivatives showed low activity levels in most organs at 24 h after injection, indicating a rapid clearance from nontumor tissue, as is especially important for blood and GRPR-positive organs such as pancreas and intestine. ^177^Lu-NeoBOMB1 showed increased activity levels in several nontumor organs at 24 h after injection, particularly in lung, liver, spleen, pancreas, intestine, and adrenals. Bone uptake was slightly enhanced for ^177^Lu-RM2, a finding that was attributed to incomplete labeling (radiochemical yield, ∼95%; chromatogram not shown) and thus free ^177^LuCl_3_. Tumor retention was comparable for all compounds except ^177^Lu-AMTG, which exhibited distinctly increased values at 24 h after injection (7.2–8.5 vs. 11.5 percentage injected dose per gram; %ID/g). Not surprisingly, ^177^Lu-AMTG showed the highest tumor-to-background ratios at 24 h after injection. The tumor-to-blood ratio of ^177^Lu-AMTG (2,702 ± 321) was almost 4 times higher than that of ^177^Lu-RM2 and ^177^Lu-AMTG2 and approximately 15 times higher than that of ^177^Lu-NeoBOMB1 (Supplemental Table 2).

**TABLE 1.** Biodistribution of ^177^Lu-RM2, ^177^Lu-NeoBOMB1, ^177^Lu-AMTG, and ^177^Lu-AMTG2 in Selected Organs at 24 Hours After Injection in PC-3 Tumor–Bearing CB17-SCID Mice (100 pmol Each)

Organ	^177^Lu-RM2	^177^Lu-NeoBOMB1	^177^Lu-AMTG	^177^Lu-AMTG competition study	^177^Lu-AMTG2	^177^Lu-AMTG2 competition study
Blood	0.012 ± 0.001	0.057 ± 0.027	0.004 ± 0.001	0.003 ± 0.000	0.011 ± 0.000	0.002 ± 0.001
Heart	0.06 ± 0.00	0.10 ± 0.03	0.02 ± 0.00	0.02 ± 0.01	0.03 ± 0.02	0.02 ± 0.01
Lung	0.10 ± 0.02	0.43 ± 0.00	0.04 ± 0.01	0.27 ± 0.15	0.05 ± 0.01	1.18 ± 1.49
Liver	0.45 ± 0.03	1.60 ± 0.62	0.14 ± 0.03	1.40 ± 0.86	0.32 ± 0.14	0.74 ± 0.44
Spleen	0.20 ± 0.02	1.94 ± 0.97	0.10 ± 0.02	2.97 ± 2.01	0.15 ± 0.07	1.06 ± 1.08
Pancreas	0.43 ± 0.06	8.48 ± 0.92	0.56 ± 0.30	0.05 ± 0.03	0.95 ± 0.14	0.07 ± 0.01
Stomach	0.19 ± 0.06	1.29 ± 0.12	0.10 ± 0.04	0.04 ± 0.02	0.12 ± 0.03	0.04 ± 0.01
Intestine	0.22 ± 0.04	0.85 ± 0.05	0.20 ± 0.10	0.27 ± 0.21	0.30 ± 0.04	0.34 ± 0.35
Kidney	1.79 ± 0.05	1.90 ± 0.72	1.16 ± 0.20	1.17 ± 0.26	1.87 ± 0.27	1.63 ± 0.44
Adrenal	0.80 ± 0.16	3.44 ± 0.25	0.46 ± 0.22	0.09 ± 0.07	0.26 ± 0.14	0.03 ± 0.02
Muscle	0.011 ± 0.011	0.010 ± 0.005	0.005 ± 0.003	0.003 ± 0.002	0.003 ± 0.003	0.003 ± 0.002
Bone	1.31 ± 0.56	0.20 ± 0.06	0.05 ± 0.02	0.05 ± 0.03	0.22 ± 0.05	0.02 ± 0.01
Tumor	8.45 ± 0.19	7.23 ± 0.91	11.45 ± 0.43	0.33 ± 0.20	7.97 ± 1.34	0.36 ± 0.25

Data are mean %ID/g ± SD (*n* = 4). Competition studies (mean ± SD, *n* = 3) were performed by coinjection of ^nat^Lu-RM2 (3.62 mg/kg).

**FIGURE 3.**
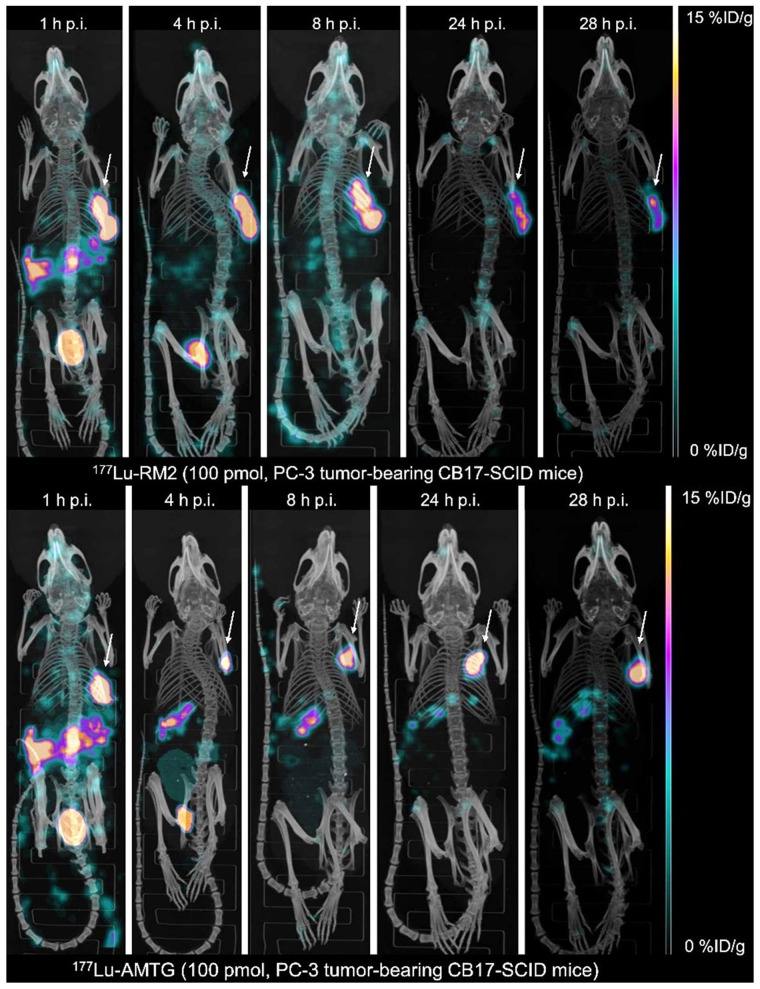
Maximum-intensity projection of PC-3 tumor–bearing CB17-SCID mice injected with ^177^Lu-RM2 and ^177^Lu-AMTG (100 pmol each). Images were acquired at 1, 4, 8, 24, and 28 h after injection into PC-3 tumors (arrows). p.i. = after injection.

Small-animal SPECT/CT studies with ^177^Lu-RM2 and ^177^Lu-AMTG at 1, 4, 8, 24, and 28 h after injection in PC-3 tumor–bearing mice revealed low background activity for both tracers at 4 h and beyond and, for ^177^Lu-AMTG, considerably higher activity in both tumor and pancreas (Fig. 3). For both tracers, specificity of tumor uptake was confirmed via competition experiments with an excess of ^nat^Lu-RM2 (Table 1; Supplemental Fig. 17).

## DISCUSSION

With regard to radioligand therapy, the 2 most promising GRPR ligands, ^68^Ga-RM2 and ^68^Ga-NeoBOMB1, present some disadvantages: ^68^Ga-RM2 suffers from rapid metabolic degradation (*17*), which is why tumor accumulation and tumor dose for ^177^Lu-RM2 are likely limited as well—especially important in the context of radioligand therapy. In contrast, ^177^Lu-NeoBOMB1, which exhibits a higher metabolic stability in vivo, shows enhanced activity retention in tumor tissue but also in blood (*19*). This characteristic results in unfavorable dosimetry and higher doses to the red bone marrow (*25*). With the aim of retaining the excellent pharmacokinetics of RM2, we substituted the unnatural amino acid α-Me-l-Trp for the metabolically less stable Gln^7^-Trp^8^ sequence of ^177^Lu-RM2 and its DOTAGA analog and compared these new ligands with ^177^Lu-RM2 and ^177^Lu-NeoBOMB1 as references.

Synthesis was easily accessible via solid-phase peptide synthesis, and complexation with ^nat^Lu or ^177^Lu proceeded quantitatively. All 4 compounds contain a similar pharmacophore typical of bombesin analogs, resulting in high affinities that were in the range of *IC*_50_ values reported for ^nat^In-RM2 (9.3 nM), several ^nat^Ga-RM26 derivatives (2.3–10.0 nM), ^nat^Ga-NeoBOMB1 (2.5 nM), and SB3 (3.5 nM) (*18**,**19**,**21**,**26*). Apart from ^nat^Lu-AMTG2, higher cellular uptake of 3-^125^I-d-Tyr^6^-MJ9, as well as slightly higher *IC*_50_ values on PC-3 cells than on T-47D cells, was observed (Supplemental Figs. 18 and 19). These findings were attributed to an increased number of receptors on PC-3 cells.

We could show that α-Me-l-Trp–for–l-Trp^8^ and DOTAGA-for-DOTA substitution had only minimal impact on GRPR affinity, lipophilicity (log*D*_7.4_), and receptor-mediated internalization, demonstrating that these modifications might allow the in vivo kinetics of ^177^Lu-RM2 to be kept almost unaffected. In contrast, higher internalization levels and lipophilicity already indicate the in vivo limitations of ^177^Lu-NeoBOMB1.

Besides retaining the favorable in vitro data of ^177^Lu-RM2, the primary aim of this study was to chemically stabilize the Gln-Trp bond to potentially improve its longtime behavior in vivo. Comparative stability studies in vitro and in vivo, as well as the resulting biodistribution profiles, substantiated our working hypothesis of addressing the major metabolic instability at the Gln-Trp site in RM2 and other bombesinlike compounds. Both ^177^Lu-AMTG and ^177^Lu-AMTG2 exhibited equal or even increased amounts of intact compound in human plasma in vitro and in murine plasma and urine in vivo, in comparison with the 2 references. For ^68^Ga-RM2 and ^177^Lu-NeoBOMB1, the fraction of intact tracer in murine blood was reported to be 55% (15 min after injection) (*27*) and 90% (30 min after injection) (*19*), respectively, which are lower than the value we determined for ^177^Lu-AMTG (30 min after injection).

Unlike Linder et al. in a stability study on ^177^Lu-AMBA (*16*), we observed fewer metabolites for each ligand after incubation in human plasma (Supplemental Fig. 14), as can be explained by the *C* terminus modifications present in each of the 4 compounds tested in this study. Popp et al. observed 1 major and 2 minor metabolites for ^68^Ga-RM2 in murine plasma at 15 min after injection (*27*), whereas we detected only 1 major and 1 minor metabolite for ^177^Lu-RM2 at 30 min after injection. This difference could be due either to our analysis method or to the effect reported by Linder et al. that the minor metabolites can be further metabolized to yield the major metabolite, the longer the circulation in vivo takes place.

**FIGURE 4.**
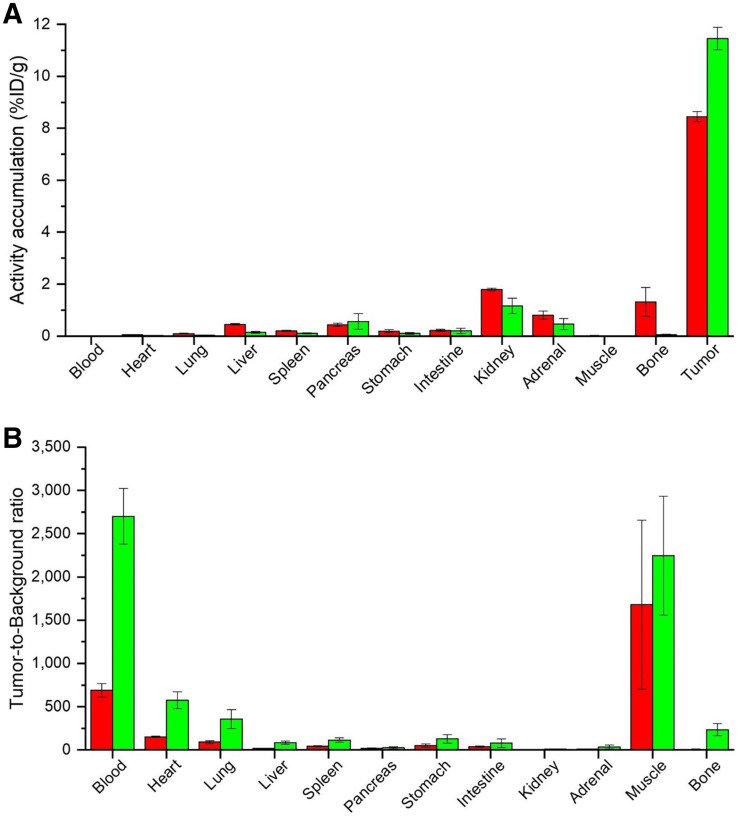
(A) Biodistribution of ^177^Lu-RM2 (red) and ^177^Lu-AMTG (green) in selected organs at 24 h after injection in PC-3 tumor–bearing CB17-SCID mice (100 pmol each). Data are %ID/g, mean ± SD (*n* = 4). (B) Tumor-to-background ratios for selected organs for ^177^Lu-RM2 (red) and ^177^Lu-AMTG (green) at 24 h after injection in PC-3 tumor–bearing CB17-SCID mice. Data are mean ± SD (*n* = 4).

Not surprisingly, increased metabolic stability observed in human and murine plasma for ^177^Lu-AMTG resulted in a 35% higher activity level in PC-3 tumor for ^177^Lu-AMTG than for ^177^Lu-RM2 at 24 h after injection (Fig. 4). Both ^177^Lu-AMTG and ^177^Lu-AMTG2 exhibited excellent clearance kinetics and thus low activity levels in nontumor organs, with the highest values obtained for the kidneys (1.2–1.9 %ID/g). Both compounds were cleared mostly intact (Supplemental Fig. 16), a finding that could favor ^177^Lu-AMTG and ^177^Lu-AMTG2 over ^177^Lu-RM2 and ^177^Lu-NeoBOMB1, as charged metabolites tend to be taken up by and retained in the kidneys. Most importantly, the activity concentration in the blood and in the GRPR-positive pancreas was low for all ^177^Lu-RM2 analogs at 24 h after injection (<0.01 and <1 %ID/g, respectively), which we considered another prerequisite for successful translation into humans.

In contrast, ^177^Lu-NeoBOMB1 displayed enhanced activity in most nontumor organs and thus the lowest tumor-to-background ratios in most organs, especially in blood, liver, spleen, pancreas, and adrenals, a finding that was also observed by other groups (*19**,**25*). The biodistribution profiles confirmed our concerns about increased lipophilicity and internalization rates. It might be speculated that retention in the GRPR-positive pancreas could be caused by a partial agonistic behavior observed in our internalization study, since GRPR agonists such as PESIN or AMBA that exhibit internalization rates of more than 25% at 1 h in vitro typically show a slow clearance from the pancreas over time (*28**,**29*).

**FIGURE 5.**
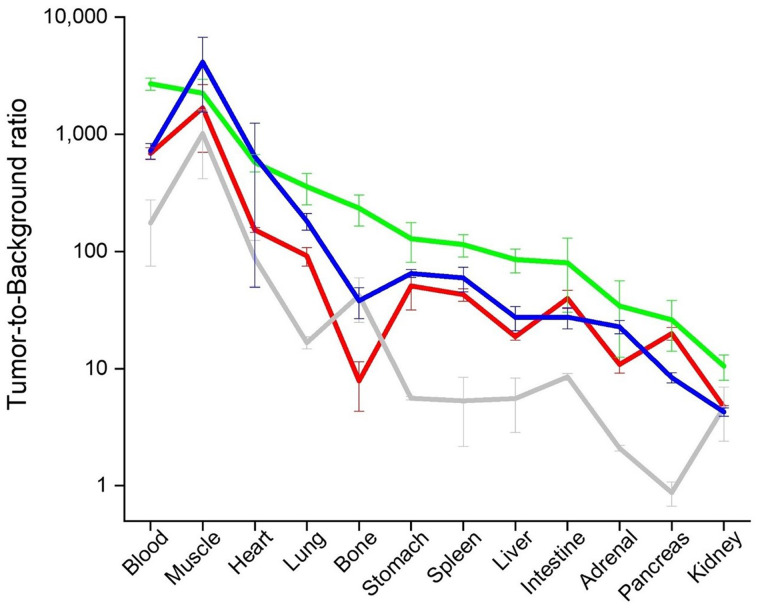
Graphic comparison of tumor-to-background ratios for selected organs for ^177^Lu-RM2 (red), ^177^Lu-NeoBOMB1 (gray), ^177^Lu-AMTG (green), and ^177^Lu-AMTG (blue). Biodistribution studies were performed at 24 h after injection in PC-3 tumor–bearing CB17-SCID mice. Data are mean ± SD (*n* = 4).

Although reduced internalization might have other causes, the high structural similarity of ^177^Lu-AMTG/^177^Lu-AMTG2 to the known GRPR antagonist ^177^Lu-RM2 and the comparably low internalization pattern observed in our studies are strong indicators of antagonistic behavior by these new compounds. This assumption is further corroborated by rapid pancreatic clearance within 24 h after injection and the resulting favorable biodistribution profiles. Evidence was also provided by small-animal SPECT/CT scans with ^177^Lu-RM2 and ^177^Lu-AMTG over time, both of which showed high tumor retention and fast clearance from nontumor organs, even the GRPR-positive pancreas. It is noteworthy that clearance from pancreas and tumor was less rapid for ^177^Lu-AMTG, confirming our hypothesis on increased metabolic stability in vivo generated by a simple modification at the Trp^8^ site. Thus, it is not surprising that tumor-to-background ratios for ^177^Lu-AMTG were highest in all organs, except for the tumor-to-muscle ratio (Fig. 5).

Regarding dose-limiting organs in the context of radioligand therapy, the excellent tumor-to-kidney and tumor-to-blood ratios make ^177^Lu-AMTG a highly attractive alternative to ^177^Lu-RM2 (*30*). In fact, ^177^Lu-AMTG seems to synergistically combine the advantages of ^177^Lu-RM2 and ^177^Lu-NeoBOMB1 regarding pharmacokinetics and stability while simultaneous offering the best GRPR affinity, both on PC-3 cells and on T-47D cells. Thus, a clinical assessment (e.g., clinical phase I study) with ^177^Lu-AMTG seems warranted.

In summary, we were able to successfully introduce an α-Me-l-Trp–for–l-Trp^8^ substitution within the pharmacophore of ^177^Lu-RM2 that not only resulted in a new tracer (^177^Lu-AMTG) with comparable affinity, internalization, and lipophilicity but also resulted in considerably improved metabolic stability. Hence, improved tumor uptake and pharmacokinetics superior to those of the parent peptide, ^177^Lu-RM2, or the second reference compound, ^177^Lu-NeoBOMB1, were observed for ^177^Lu-AMTG. A noteworthy finding is that improved metabolic stability was achieved without coadministration of peptidase inhibitors (*21*), such as phosphoramidon; this finding could facilitate clinical translation. It seems legitimate to conclude that other bombesin derivatives published in recent years, which have been modified at the *N* or *C* terminus but not at the unstable dipeptide sequence Gln^7^-Trp^8^ (*20**–**22*), would also benefit from an α-Me-l-Trp–for–l-Trp^8^ substitution. Nevertheless, studies on prostate and breast cancer patients have to be performed to show whether these promising preclinical results are reflected on a clinical level.

## CONCLUSION

We could demonstrate that the new ^177^Lu-RM2 derivative ^177^Lu-AMTG offers better overall preclinical performance than ^177^Lu-RM2 or ^177^Lu-NeoBOMB1. On the basis of these results, a clinical translation of ^177^Lu-AMTG is highly recommended to assess a potential improved therapeutic value for radioligand therapy of GRPR-expressing malignancies, such as prostate and breast cancer. In addition, we expect that substitution of L-amino acids by their corresponding α-alkyl-l-amino acid analogs could also be a valuable approach to stabilize the pharmacophore of other peptidic ligands that suffer from insufficient stability in vivo.

## DISCLOSURE

A patent application has been filed on modified GRPR-targeted ligands, including AMTG, with Thomas Günther and Hans-Jürgen Wester as inventors. Parts of this study were funded by the SFB 824 (DFG Sonderforschungsbereich 824, project Z [Hans-Jürgen Wester]) from the Deutsche Forschungsgemeinschaft, Bonn, Germany. Hans-Jürgen Wester is founder and shareholder of Scintomics GmbH, Munich, Germany. No other potential conflict of interest relevant to this article was reported.

## Notes

KEY POINTS **QUESTION:** Is it possible to overcome the major limitation of ^177^Lu-RM2, its metabolic instability, with regard to future GRPR-targeted radioligand therapy by means of a tiny modification without negatively influencing overall pharmacokinetics? **PERTINENT FINDINGS:** Substitution of l-Trp^8^ by α-Me-l-Trp^8^ in the pharmacophoric sequence of ^177^Lu-RM2 retains the favorable tracer pharmacokinetics while enhancing metabolic stability, making ^177^Lu-AMTG a highly promising novel GRPR-targeted radiopharmaceutical for radioligand therapy. **IMPLICATIONS FOR PATIENT CARE:** Although the clinical value of ^177^Lu-AMTG has to be determined in clinical studies, this study could open new possibilities for complementary treatment of prostate and breast cancer.
